# PortionControl@HOME: Results of a Randomized Controlled Trial Evaluating the Effect of a Multi-Component Portion Size Intervention on Portion Control Behavior and Body Mass Index

**DOI:** 10.1007/s12160-014-9637-4

**Published:** 2014-08-21

**Authors:** Maartje P. Poelman, Emely de Vet, Elizabeth Velema, Michiel R. de Boer, Jacob C. Seidell, Ingrid H. M. Steenhuis

**Affiliations:** 1 Department of Health Sciences and EMGO+ Institute for Health and Care Research, VU University Amsterdam, De Boelelaan 1085, 1081 HV Amsterdam, The Netherlands; 2 Department of social sciences, Sub-department Communication, Philosophy and Technology: Centre for Integrative Development, Chairgroup Strategic Communication, Wageningen University and Research Centre, Wageningen, The Netherlands

**Keywords:** Portion size, Intervention, Obesity, Weight loss, Portion control, Self-regulation

## Abstract

**Background:**

Food portion sizes influence energy intake.

**Purpose:**

The purpose of this paper is to determine effectiveness of the “PortionControl@HOME” intervention on body mass index and portion control behavior.

**Methods:**

A randomized controlled trial among 278 overweight and obese participants was conducted. PortionControl@HOME aimed to increase: portion size awareness, portion control behavior, portion control cooking skills, and to create a home environment favoring portion control.

**Results:**

Intention-to-treat multi-level regression analysis indicated statistically significant effects of the intervention on portion control behavior at 3, 6, and 12 months follow-up. The effect on body mass index was significant only at 3 months follow-up and when outliers (*n* = 3) were excluded (*B* = −0.45; 95 %CI = −0.88 to −0.04). The intervention effect on body mass index was mediated by portion control behavior.

**Conclusions:**

The intervention improves portion control behavior, which in turn influence body mass index. Once the intervention ceased, sustained effects on body mass index were no longer evident. (Current-Controlled-Trials ISRCTN12363482).

**Electronic supplementary material:**

The online version of this article (doi:10.1007/s12160-014-9637-4) contains supplementary material, which is available to authorized users.

## Introduction

Globally, obesity has more than doubled since 1980 and has become a public health crisis. Worldwide, 35 % of adults are overweight and 11 % are obese [1]. The high prevalence of overweight and obesity is associated with an elevated incidence of co-morbid conditions, such as diabetes mellitus type II [2] .

Overweight and obesity develop when energy intake continually exceed energy expenditure. Food portion sizes strongly influence energy intake. Research has shown that when people are offered larger portions their energy intake increases [e.g., 3, 4]. This portion size effect has been demonstrated for a variety of foods, complete daily menus, and even for foods with a perceived unfavorable taste [5, 6]. For example, individuals consumed 30 % more energy when offered a large portion (1,000 g) of macaroni and cheese compared to when they were offered a “small” portion (500 g) [4]. The effects of large food portions on energy intake can persist over several days, with no indication of full compensation [7, 8]. Since the 1970s, portion sizes of foods eaten inside and outside the home have increased considerably, and larger food portions are readily and widely available [9–11]. Given the pervasiveness of large portions in the current food environment and the evidence regarding the influence of portion size on energy intake, food portions are widely accepted as contributing to the global obesity epidemic [12, 13].

A range of environmental interventions have been proposed to reverse the trend towards large portions and the associated higher energy intake [14], many of which have been evaluated for effectiveness [15–22]. For example, a study in worksite cafeterias where small portions of a hot meal were introduced alongside standard (large) portions (approximately 2/3 of the weight of the standard portion) showed that approximately 10 % of the consumers replaced the regular meal with a small meal [15]. Although this experiment did not determine the effect on actual energy intake, other studies assessing the effect of smaller portions on energy intake have shown mixed effects. While one study demonstrated that a portion size reduction of 25 % led to a decrease in energy intake in a laboratory setting [8], limiting portion sizes in a university dining facility did not impact visitor’s energy intake [20]. Interventions regarding portion size labeling have shown limited effects on portion size selection or energy intake [17, 19, 23], although one study found that non-dieters ate significantly less of a snack food when confronted with dual-column labeling, which contained both single serving and entire package nutritional information [22]. Removing value size pricing also had little effect on the selection of smaller food portions or decreased energy intakes. Nevertheless, it was emphasized that repeated exposure of an absent value size pricing may be required in order to achieve an effect [18, 21].

While environmental strategies to decrease portion sizes seem to be the most common sense approach to impact population level energy intake, the effects of these strategies on consumption behavior have been mixed. In addition, the feasibility of such environmental interventions needs to be considered in the context of the many parties or organizations (i.e., industry, government) that would be involved, many of whom may have conflicting goals in terms of public health. Therefore, it cannot be ignored that tension might exist between effective interventions on consumption behavior and the feasibility in real-life settings. Although an ongoing effort to develop effective and feasible environmental portion size interventions is necessary, an alternative solution might be to improve peoples’ ability to control and maintain adequate portion size selection and intake, thereby dealing with environmental stimuli to consume large food portions.

Already, some educational interventions to help people to cope with the availability of large food portions exist. Most educational interventions have focused on decreasing individuals’ experience of portion distortion [24–28], which refers to the phenomenon that people perceive larger portions to be an appropriate amount to eat on a single eating occasion [29]. Most of these educational interventions had positive effects on improving awareness, estimation skills or knowledge about portion sizes, although none of the studies assessed the impact on body mass [24–28].

Although knowledge and awareness about food portions are important prerequisites for behavioral change, these basics are insufficient to achieve changes in actual consumption [30, 31]. Self-regulation of portion sizes selection and intake, and the ability to cope with the supersized food environment are also required [14, 32]. Self-regulation refers to all efforts to steer attention, emotions, and behaviors to reach beneficial long-term goals (i.e., weight loss), even when there are short-term temptations (i.e., a nice cookie) or conflicting long-term goals [33]. Therefore, self-regulation strategies to control portion size selection and intake are likely to be useful for people to obtain. The home food environment is the place where the retail food environment comes together with actual food intake [34, 35] and is an important place that influences portion size regulation [36] and dietary intake [37]. Helping individuals to create a supportive home food environment can be another important target in assisting adequate portion control behavior [37].

Therefore, the aim of the present study was to examine the effect of the multi-component educational intervention “PortionControl@HOME” on body mass index and portion control behavior. The hypotheses were that compared to a control group, body mass index would be more favorable at follow-up for the intervention group, and participants in the intervention group would employ portion control strategies more frequently. It was also hypothesized that the impact of the intervention on change in body mass index would be mediated through an increased use in portion control strategies.

## Methods

### Participants

The study was conducted according to the ethical standards declared in the Helsinki Declaration of 1975, as revised in 2000, approved by the Medical Ethics Committee of the VU Medical Center, and registered with controlled-trials.com (ISRCTN12363482). From October to December 2011, participants were recruited from six municipalities in the Netherlands in an urbanized area 21–45 km from Amsterdam. Dutch adults were eligible to participate if they met the following criteria: (1) body mass index above 25 kg/m^2^, (2) aged between 18 and 60 years, (3) residing in or within a distance of 15 km of one of the participating six municipalities, (4) were the nutritional gatekeeper of the family (the person of the family who has the biggest food influence, defined by being responsible for doing groceries or making dinner) [38], and (5) only one person per address could participate. Participants were excluded when they reported having or having had one of the following co-morbidities associated with overweight and obesity: (1) diabetes mellitus, (2) cardiovascular diseases, (3) cancer or (4) clinical depression. Moreover, participants that reported to be on a diet visited a dietician or reported to be or have been in an intensive weight loss treatment within the past 6 months were excluded from participation. Finally, females who reported being pregnant or planning to become pregnant were excluded.

**Fig. 1:**
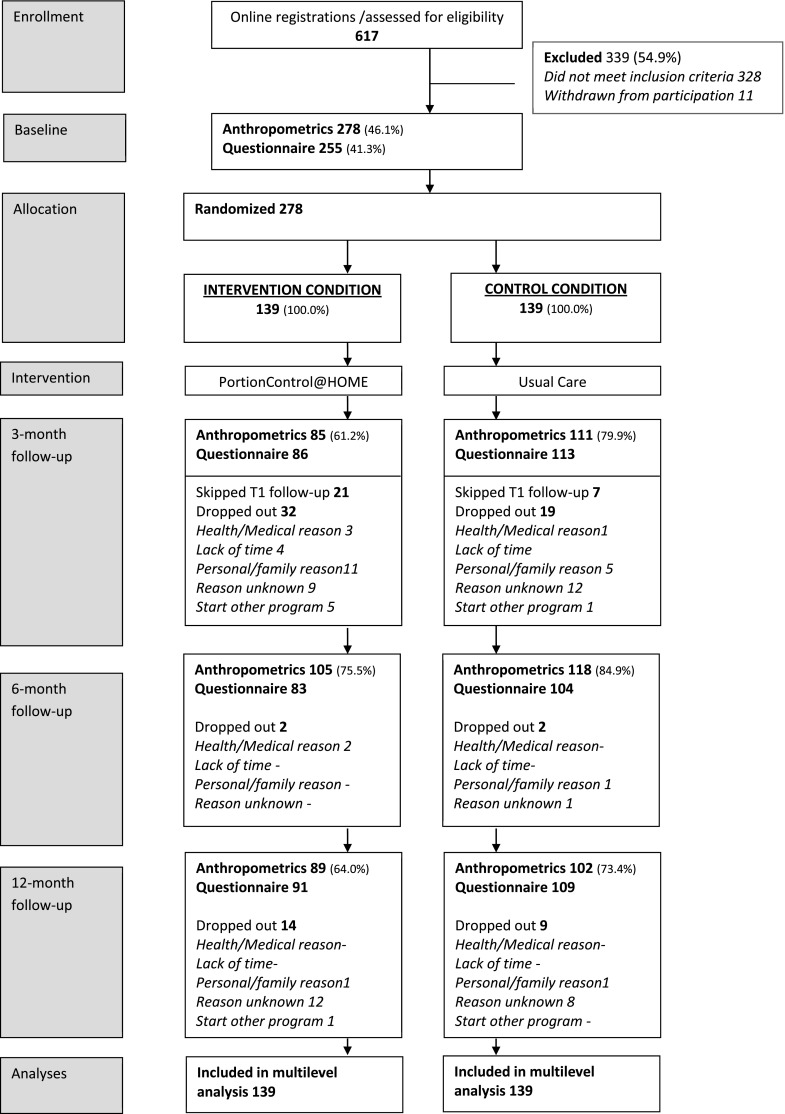
Flow of recruitment, randomization and follow-up of the study participants

Several strategies were used to recruit study participants. First, willing general practitioners (35 of the contacted 73 professionals (47.9 %)) and physiotherapists (28 of the contacted 55 professionals, (50.9 %)) in the six municipalities distributed an information letter to overweight or obese patients that visited their practice during the recruitment period. This information letter referred potential participants to the study website. Second, flyers and posters referring to the study website were placed in pharmacies (40 locations), public facilities (39 locations) and in the waiting rooms of willing physiotherapists and general practitioners. Finally, participants were recruited through advertisements in local newspapers (in all municipalities) and by messages by local radio stations (in two municipalities). Adults willing to participate had to visit the study website to register themselves for the study and were asked to complete the online application form. All registrations were screened for eligibility by the research team (Fig. 1).

### Design

A two-arm, parallel design, randomized controlled trial (RCT) was conducted between January 2012 and February 2013. After baseline measurements, all participants were randomized at the same time to either the intervention “PortionControl@HOME” group or to a wait-list control group. In each municipality, participants were randomly assigned separately in order to ensure an equal balance in the number of participants per group. Randomization lists were generated with standard statistical computer software (IBM SPSS Statistics 20.0). Based on the randomization list, the researcher (M.P.P) allocated subjects to one of the groups. Due to the nature of the intervention, it was not possible to blind participants to their allocated condition.

### Procedures

Eligible participants completed assessments at baseline (T0, prior to randomization), and at 3 (T1), 6 (T2), and 12 (T3) months of follow-up (Fig. 1). All procedures followed were in accordance with ethical standards of the responsible committee on human experimentation and prior to the baseline measurements, written informed consent was obtained. Baseline characteristics (age, sex, educational level, nationality) were assessed by self-report at enrollment via the online application form and during the baseline measurement (T0). Objective measures of body weight and height were collected during home visits by the researchers (E.V. and M.P.P) at baseline (T0) and 6 months follow-up (T2). Data of body weight were obtained by self-reported data at 3 months (T1) and 12 months (T3) follow-up. Data on portion control behavior was collected by means of questionnaires at baseline and each follow-up assessment. Intervention adherence among intervention group participants was self-reported at 3 months follow-up (T1) or objectively measured by the research team when possible. Dieting behavior was assessed by means of one item in the questionnaires at 3 (T1), 6 (T2), and 12 months (T3) follow-up. All participants received incentives to prevent drop out from the study in the form of gifts (i.e., tea-box presents, UEFA Euro cup 2012 gadgets) and a reward coupon (€ 10) for completing the assessments.

### Intervention Overview

The intervention group received the PortionControl@HOME intervention program over 3 months (between T0 and T1). PortionControl@HOME was developed to stimulate adequate portion control behavior in order to decrease energy intake. The intervention was theory-based and consisted of the following four elements that were developed, pilot tested and reiterated prior to implementation: (1) PortionSize@warenessTool [25]. (2) Portion Control Strategies [32]. (3) Portion control cooking class. (4) Portion control Home-Screener. After completion of the intervention (between T1 (3 months follow-up)) and T3 (12 months follow-up) the intervention group received three online portion control boosters vie email repeating the PortionControl@HOME content. The theoretical underpinning and a comprehensive description of the intervention elements are provided in the first two columns of Table 1 in the Electronic Supplementary Material.

### Control Condition

The wait-list control group continued with their normal behavior and received the program—without the cooking class—after the 12-month assessments.

### Sample Size

An a priori sample size estimate indicated 284 participants (142 per group) would be sufficient to detect a difference of 1 body mass index point with a two-sided 5 % significance level and a power of 80 %, given an anticipated dropout rate of 10 %.

### Measures

The primary outcome was body mass index assessed at 3, 6, and 12 months. Secondary outcomes included the use of the portion control strategies, intervention adherence and evaluation, and additional dieting behavior.

#### Baseline Characteristics

Age, sex, nationality, and educational level were assessed by self-report. Education was based on the highest qualification attained and was classified in three groups of educational level: low (“those with less than secondary school or an A-level certificate”), middle (“those with A-levels or Dutch A-level equivalent (VWO) graduation certificate”) and high (“those with polytechnic or university degrees”).

#### Body Mass Index

At baseline (T0), weight was measured using two different scales: a professional one (the Marsden MPMS-250 digital scale, Oxfordshire, UK) and the participant’s scale, in light clothes and with shoes removed. Measurements were highly correlated (regression coefficient = 0.99; intercept = -0.10), indicating that both scales yield largely similar results. At T2, the weight was also objectively measured using the professional scale, during a home visit from the researchers. At T1 and T3, participants were asked again to weigh themselves. Height was measured at baseline to the nearest millimeter with a stadiometer (Seca 214, Hamburg, Germany). Body mass index was calculated as weight (kg)/height (m)^2^.

#### Portion Control Behavior

Self-administered portion control behavior was measured using a 32-item questionnaire that assessed the strategies incorporated in the PortionControl@HOME intervention program. Participants indicated their use of the strategies on a five-point Likert scale ranging from 1 (almost never) to 5 (almost always). A mean score of the 32 items was computed to reflect general use of portion control strategies (T0 Cronbach’s *α* = 0.78). This questionnaire has been used before and is described in more detail elsewhere [32].

#### Dieting Behavior

To account for any possible confounding effects on weight change and to rule out alternative explanations for weight loss, dieting behavior was assessed by one item (e.g., “Did you follow a popular diet or followed a weight loss treatment during the past three months period?”) followed by seven response options ranging from “never”- “yes, over the past three months”. Answers were categorized by “never” “shorter than one month” and “1 month or more” and percentages for each group were calculated.

#### Intervention Adherence

Intervention group participants self-reported their use of the educational book (“Did you read the educational book”? ‘Yes, completely’/‘Yes, partly’/‘No, not at all’). The use of the Home-Screener was determined by two questions: (a) “Did you complete the screening as proposed by the Home-Screener” ‘Yes, completely’/‘Yes, partly’/‘No’ and (b) “Did you read the advice regarding your home food environment provided by the Home-Screener”? ‘Yes, all advice’/‘yes, some advice’/‘No, not any advice’) at 3 months follow-up (T1). Fidelity of the cooking class and website was assessed by recording attendance with cooking classes (ranging from zero to 3 classes) or using the PortionSize@awarenessTool (logged-in on the website: yes/no). Also the fidelity of the action planning, coping planning, and self-monitoring exercises/assignments were determined by items asking the participants if they fulfilled the assignment ‘yes, in mind’/‘yes, wrote down on the assessment form’/‘no, not at all’.

### Statistical Analyses

Statistical analyses were conducted using standard statistical computer software (IBM SPSS Statistics 20.0). All statistical tests were two-tailed and a 5 % significance level maintained throughout the analyses.

Descriptive statistics were used to characterize the intervention and control group at baseline. Moreover, descriptive statistics were used to identify dieting behavior of all participants and the overall intervention adherence of the intervention group participants.

A multi-level regression analysis on the principle of intention-to-treat (ITT) was conducted, using the data collected from all randomized participants. First, crude analyses including adjustment for baseline values of the outcomes were conducted. Second, based on previous studies indicating associations between portion control behavior and socio-demographic characteristics we adjusted for age, sex, and educational level additionally [32]. In a third model, dieting behavior was also entered as a covariate in the adjusted analysis of the primary outcome. All models included time and an interaction term between time and the intervention variable in order to assess time specific intervention effects. Analysis including and excluding the outliers (body mass index change >3× SD above or below mean body mass index change between T0 and T2 (objectively measured data)) were performed to whether the intervention effect was robust against these outliers in the data. Similar analyses were conducted for portion control behavior.

A mediation analysis was conducted to determine whether portion control behavior mediated the potential intervention effect on body mass index [39]. To achieve this, the association between “group” and “portion control behavior” was first assessed. Second, the association between “portion control behavior” and “body mass index” was ascertained. Third, the association between “group” and “body mass index” was examined. Finally, the association between “group” and “body mass index” adjusted for the variable “portion control behavior” was established. A Sobel test [40] was conducted to determine if potential mediation was statistically significant.

## Results

### Response

Figure 1 shows a flow chart of the number of screened potential participants, recruitment, and follow-up. A total of 617 individuals completed the online registration, of which 289 fulfilled the inclusion criteria. Reporting “suffer from or having a history with one of the co-morbidities associated with overweight and obesity” was the most important reason why participants were excluded from participation. When contacted by the research team, 11 participants were ineligible prior to randomization (e.g., pregnant, refuse to participate, failed to contact). Therefore, a total number of 278 participants were randomized into the intervention or control group. At 3 month (T1) follow-up, 85 (61.2 %) and 111 (79.9 %) participants in the intervention and control condition, respectively, returned the questionnaires with weight related measures. Reasons for missing data included questionnaires not being completed and returned, or participants requesting dropout. At 6 months follow-up (T2), a larger sample of individuals completed the measurements compared to 3 months follow-up T1, probably due to the fact that they were visited at home by the researchers. During this measurement, a total of 105 (75.5 %) individuals in the intervention group and 118 (84.9 %) individuals in the control group participated. At 12 months follow-up (T3), 89 (64.0 %) and 102 (73.4 %) participants completed the weight related measures themselves. Logistic regression analysis determining dropout (yes/no) including the variables age, sex, educational level, and group-allocation indicated that allocation to the intervention group was significantly associated with higher dropout rates during the study (odds ratio (OR) = 2.2, *p* = 0.013).

### Baseline Characteristics

A total of 278 participants completed the baseline measurements. Participants were on average 45.7 (SD = 9.2) years old, the majority were female (84.5 %) and obese (65.1 %). Almost all participants (96.8 %) had a Dutch nationality (other 3.2 %, e.g., Surinam, French, German) and 42.7 % had a high educational level (Table 1).

**Table 1:** Baseline characteristics of the participants allocated to the intervention and control condition

Group characteristics		All	Intervention group	Control group
	*N*	278	139	139
Age	Mean (SD)	45.65 (9.20)	45.87 (9.22)	45.42 (9.21)
Sex	female % (*n*)	84.5 (235)	84.9 (118)	84.2 % (117)
Education	% low	20.9	24.8	17.2
	% medium	36.4	32.0	40.6
	% high	42.7	42.2	43.2
Weight (kg)	Mean (SD)	94.11 (15.8)	94.95 (15.37)	93.27 (16.20)
Body mass index (kg/m^2^)	Mean (SD)	32.4 (4.8)	32.86 (4.95)	32.00 (4.57)
Body mass index 25 ≤ 30	% (*n*)	34.9 (97)	31.7 (44)	38.1 % (53)
Body mass index >30	% (*n*)	65.1 (181)	68.3 (95)	61.9 % (86)

### Dieting Behavior

Participants in both the intervention and the control group reported to be on a diet during the follow-up measurements. Participants in the intervention and. the control condition were on a diet for more than 1 month in the following proportions: T1, 7.0 % vs. 15.9 %; T2, 8.5 % vs. 15.5 %; and at T3, 5.0 % vs. 7.5 %. At 3 months follow-up significantly more participants in the control group were on a popular diet for a longer period (>1 month) than participants in the intervention condition (*X* ^2^ = 5.97, *df* = 2, *p* = 0.05).

### Body Mass Index

Overall, mean body mass index decreased in both groups between baseline and T1 (intervention 2 points and control 1 point, Table 2) but increased again between T1 and T3, although mean body mass index remained below the baseline values. Mixed model analysis showed that at 3 months, the estimated difference between the intervention group and the control group in the fully adjusted model (corrected for demographic variables and dieting behavior) was −0.42 (95 %CI = −0.91 to 0.07, Table 3). This difference was more pronounced and statistically significant when outliers (*n* = 3) were excluded from the analyses (*B* = −0.45; 95 %CI = −0.88 to −0.04). Differences between both groups were smaller at 6 months follow-up (*B* = −0.13; 95 %CI = −0.63 to 0.37) and absent at 12 months follow-up (*B* = −0.03; 95 %CI = −0.53 to 0.47) (Table 3).

**Table 2:** Descriptive statistics Body Mass Index and Portion Control Behavior of the complete cases in the intervention group and control group at baseline (T0) and 3 (T1), 6 (T2), and 12 (T3) months follow-up

	*n*	T0	*n*	T1	*n*	T2	*n*	T3
Body mass index^a^								
Intervention group	139	32.86 (4.95)	85	30.88 (4.73)	105	32.15 (5.07)	89	31.45 (4.96)
Control group	139	32.00 (4.57)	111	30.95 (4.69)	118	31.40 (4.75)	102	30.84 (4.73)
Portion control behavior^b^								
Intervention group	127	3.24 (0.46)	86	3.73 (0.48)	83	3.68 (0.50)	91	3.77 (0.48)
Control group	126	3.21 (0.49)	113	3.36 (0.50)	104	3.40 (0.51)	109	3.42 (0.42)

^a^T0 and T2 represent the objectively measured body mass index and T1 and T3 represent the subjectively measured Body Mass Index

^b^Portion control behavior measured on a five-point Likert scale

**Table 3:** Time specific intervention effects on body mass index and portion control behavior at 3, 6, and 12 months follow-up

	Body mass index (kg/m^2^)						Portion control behavior					
	Body mass index			Body mass index without outliers^a^			Portion control behavior			Portion control behavior without outliers^a^		
	Estimates of fixed effects coefficients between groups (95 % CI)						Estimates of fixed effects coefficient between groups (95 % CI)					
3 months follow-up (T1)												
Crude model^b^	−0.29	(−0.77	0.20)	−0.37	(−0.79	0.04)	0.32	(0.22	0.42)	0.31	(0.22	0.41)
Adjusted model 1^c^	−0.32	(−0.83	0.18)	−0.40	(−0.83	0.03)	0.31	(0.21	0.42)	0.31	(0.21	0.40)
Adjusted model 2^d^	−0.42	(−0.91	0.07)	−0.45	(−0.88	−0.04)*	0.33	(0.23	0.43)	0.32	(0.22	0.42)
6 months follow-up (T2)												
Crude model^b^	0.035	(−0.44	0.51)	−0.12	(−0.52	0.29)	0.29	(0.19	0.40)	0.26	(0.16	0.36)
Adjusted model 1^c^	0.002	(−0.49	0.49)	−0.14	(0.56	0.28)	0.29	(0.18	0.39)	0.26	(0.16	0.36)
Adjusted model 2^d^	−0.13	(−0.63	0.37)	−0.23	(0.66	0.19)	0.30	(0.19	0.40)	0.27	(0.17	0.36)
12 months follow-up (T3)												
Crude model^b^	0.04	(−0.46	0.53)	−0.16	(−0.58	0.26)	0.35	(0.25	0.45)	0.32	(0.23	0.42)
Adjusted model 1^c^	−0.01	(−0.51	0.49)	−0.20	(−0.63	0.23)	0.34	(0.24	0.44)	0.32	(0.22	0.41)
Adjusted model 2^d^	−0.03	(−0.53	0.47)	−0.19	(−0.62	0.24)	0.35	(0.24	0.45)	0.32	(0.22	0.42)

*p ≤ 0.05

^a^Outliers are defines as 3× larger or smaller than the standard deviation of the mean difference between T0 and T2: body mass index (±5.52 body mass index points): 3 outliers ((1) −5.52; (2) −12.73; (3) −10.56 body mass index points). Portion control behavior (±1.20 points) : 4 outliers

^b^Adjusted for baseline body mass index

^c^Adjusted for baseline body mass index, age, sex and educational level

^d^Adjusted for baseline body mass index, corrected for age, sex and educational level + dieting behavior in the period prior to the measurement

### Portion Control Behavior

Table 2 shows that mean portion control behavior for the intervention group at T1 increased by 0.49 points (on a five-point Likert scale) and that this stabilized over the follow-up period. At follow-up, the estimated differences between the intervention and the control group were 0.33 (95 %CI = 0.23 to 0.43), 0.30 (95 %CI = 0.19 to 0.40) and 0.35 (95 %CI = 0.24 to 0.45) at 3, 6, and 12 months, respectively. These differences were less evident when outliers were excluded from the analyses, although differences between groups remained statistically significant (Table 3).

### Mediation Analysis

**Fig. 2:**
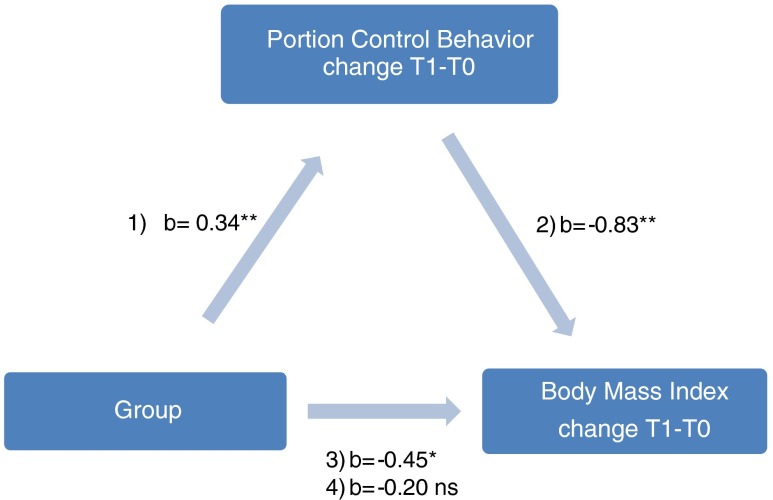
Mediation analysis of group-condition, portion control behavior and body mass index at 3 months follow-up. *ns* non significant; *Change T1 − T0* differences between 3 months follow-up and baseline measurements for portion control behavior or body mass index; *Group* intervention compared to the control condition; *1, 2, 3* Regression coefficient between the variables; *4* regression coefficient between variables after correction for portion control behavior change T1 − T0. *p < .05; **p < .01

Because the mixed model analyses only indicated an effect of the intervention on body mass index change at 3 months follow-up, a mediation analysis was only conducted for this time (baseline to 3-month follow-up). Results showed a statistically significant positive association between group-allocation (intervention) and portion control behavior (*b* = 0.34, *p* < 0.01), as well as a negative association between portion control behavior and body mass index (*b* = −0.83 *p* < 0.01). The intervention was also statistically significantly associated with a decrease in body mass index (*b* = −0.45 *p* < 0.05). In the final step, when portion control behavior was added as covariate, the effect of the intervention was attenuated (*b* = −0.20 *p* = 0.35), indicating the intervention effect was mediated by portion control behavior. The Sobel test indicated that portion control behavior significantly mediated the intervention effect on body mass index (*p* < 0.001, Fig. 2).

### Intervention Adherence and Evaluation

Of all 139 intervention group participants, 126 (90.4 %) logged on to the website aimed at portion size awareness and 56 (40.3 %) attended all three cooking class meetings. A total of 34 (24.5 %) and 21 (15.1 %) participants attended two or one cooking class, respectively. Twenty-eight participants (20.1 %) did not attend any cooking class. Ten participants (7.2 %) did not attend one of the cooking classes and did not visit the website. Of the 86 intervention participants that completed the follow-up questionnaires at T1, 55 (64.0 %) and 29 (33.7 %) reported that they had read the educational book completely or partly, respectively. Moreover, 48.2 % reported to have filled-out the Home-Screener completely whereas 75.3 % of the participants reported to have read all advice presented in the Home-Screener. The fidelity of the assignments (both in mind or writing down on the assessment form) regarding action planning (64.3 %) was moderately higher than the assignments regarding coping planning (42.8 %) and self-monitoring (31.4 %, Table 1 in the Electronic Supplementary Material).

## Discussion

The PortionControl@HOME study aimed to examine the effects of a multi-component educational intervention aimed at portion size on portion control behavior and weight change in overweight and obese participants. In partial support of the hypothesis, the intervention group reported a greater weight loss compared to the control group after completing the intervention (T1), although this difference (−0.45 body mass index points) was only statistically significant when three outliers were removed from the analysis. The effect of the intervention was not sustained at 6 and 12 months follow-up. Compared to the control group, the intervention group reported a significantly higher increase in self-reported portion control behavior. Further analysis indicated that the effect of the intervention on weight loss at 3 months was mediated by portion control behavior. Collectively, these results suggest that the PortionControl@HOME intervention can be useful to improve portion control behavior and initial weight loss; however additional efforts and more intensive behavioral supports are needed to strengthen and prolong the intervention effect. These findings are consistent with previous notions of positive effects on weight loss during or directly after interventions (initial change) but the disappearance of the effects once the intervention was terminated (failure of maintenance) [41].

Several explanations for the temporary intervention effect are proposed. First, the results indicated that a minority of the individuals completed the coping planning and self-monitoring assignments (with 42.8 % and 31.4 % respectively), although these strategies have been proposed as important in behavioral change [42, 43]. Moreover, while the intervention was based on behavior change theories (e.g., the health action process approach), additional behavioral change theories (i.e., dual process theory) may be needed to strengthen the intervention effects. Second, the duration of the intervention (3 months) may have been of insufficient duration to achieve adequate and prolonged weight loss among the present study population. Also the inclusion of the booster sessions had no added value, which is consistent with previous studies [44, 45]. It can also be suggested that the current intervention is more suitable for weight gain prevention. For example, the intervention could target weight maintenance among individuals with a body mass index slightly below 25. The aim of the intervention would then be to prevent overweight. However, it can be questioned if this group with a healthy body weight is motivated to participate in such a comprehensive intervention program and future studies are needed to confirm this assumption. A third explanation for the temporary effect on body mass index change might be the absence of the incorporation of relapse prevention strategies in the program. A future portion control program should also focus on relapse prevention, in order to prevent individuals from falling back to their old dietary pattern [41]. Practical portion control relapse prevention strategies that could be added to a prolonged program are: (1) The identification of personal high-risk situations for failures and lapses; (2) The practicing of coping with real-life high-risk situations; (3) The identification and use of problem-solving techniques (4) The training of using cognitive-coping strategies; or (5) The planning for a long-term portion control life style to maintain or lose weight. It is important that participants develop the mastery of such strategies that accompany the difficulties they experience to maintain their portion control behavior [46, 47].

Another direction to achieve sustainable results is to combine the educational intervention with environmental efforts aiming to create a more “portion-friendly” choice environment. The importance of combining both public health actions was demonstrated in recent research, which revealed that using self-regulation strategies weakened the effect of the food-rich environment but did not eliminate the impact of this unfavorable food environment [48]. Environmental efforts that could support portion control behavior might encourage portion sizes to align with nutritional guidelines, or could encourage reference food portions to become the default option. Although it was emphasized in the introduction that both feasible and effective environmental efforts might be difficult to achieve, ongoing research into the feasibility of such strategies and the combined effects of educational and environmental interventions is needed.

It is important to highlight that many people were interested in the intervention. A total of 620 individuals signed up for participation in the study. This suggested that overweight and obese participants are motivated to participate in behavior change interventions. A previous study among obese individuals supported this finding and showed that obese adults preferred non-commercial interventions to improve lifestyle above programs that were focused on weight loss only [49]. Moreover, our study suggested that offering the PortionControl@HOME intervention may reduce the interest in popular or “quick fix” diets, which was illustrated by the finding that less intervention group participants engaged in such diets during the intervention period.

Intervention group participants’ initial use of the program-elements was high and the use of each intervention-element (website, book, cooking class, and home-screener) ranged between 48.8 and 98.8 %. However, the fulfillment of the individual assignments as recommended by the program was much lower. Unfortunately, the loss to follow-up in the intervention group was significantly higher than in the wait-list control condition. This was probably due to the fact that the intervention group participants already completed the PortionControl@HOME program and no incentives to complete the measures were left after the intervention period. Moreover, the actual dropout (31.3 %) was higher than expected when designing the trial (10 %).

Some strengths and limitations need to be discussed. Strengths include the multi-component intervention, the pilot-testing of the elements before implementation and the use of a randomized controlled trial with intention-to-treat analysis. Limitations of the study included the amount of missing data and the partly subjectively measured body mass index [50, 51]. Self-reported body mass index has been associated with underreporting of actual weight, which in turn may be associated with an overestimation of weight loss in this study [51]. However, it was assumed that both the control and intervention group would underreport their weight to the same extent. In addition, self-reported weight and objectively measured weight was highly correlated at T0. Therefore, self-reported body mass index was regarded a valid alternative to measured body mass index.

Randomized controlled trials (RCTs) are considered to be the gold standard for determining the efficacy of interventions and we mention it as one of the strengths of our study. However, recently, it has been questioned whether a standard RCT design is the most appropriate for assessing weight loss interventions (or other lifestyle or policy programs) that are compared to usual care [52]. This is because control group participants may be motivated to change behavior throughout the study period, and thus does not truly reflect a “usual care comparison”. Moreover, behavioral weight loss intervention trials cannot be blinded or compared to a placebo control condition, which means participants in the control group are aware of their allocated condition. Considering the fact that the use of the portion control strategies were ascertained almost literally in the questionnaires (i.e. the portion control strategy “when preparing a meal, don’t snack on the ingredients” corresponded to the item in the questionnaire: “When preparing a meal, I snack on the ingredients”) the control group could have been contaminated with the portion control strategies as provided by the intervention program. When designing future studies to evaluate weight loss interventions, new designs (for example cohort randomized controlled trials) should be considered.

## Conclusion

The results of this study suggest that the intervention improved portion control behavior. Furthermore, the intervention effect, mediated by portion control behavior, may have favorable effects on weight at 3 months follow-up immediately after the intervention but not at 6 or 12 months. Therefore, additional efforts or prolonged programs are needed to maintain the effectiveness. Future research should identify effective supplemental intervention strategies and should examine the effect of portion control programs in combination with efforts that create a more “portion-friendly” choice environment.

## Electronic Supplementary Material

Below is the link to the electronic supplementary material.

ESM 1 (DOCX 31 kb)
