# Supplementary material for: PortionControl@HOME: Results of a Randomized Controlled Trial Evaluating the Effect of a Multi-Component Portion Size Intervention on Portion Control Behavior and Body Mass Index
Source: Ann Behav Med. 2014 Aug 21;49(1):18–28. doi: 10.1007/s12160-014-9637-4 (PMC4335123; doi:10.1007/s12160-014-9637-4)
Supplement: Supplementary file 1 — (DOCX 31 kb) [file 12160_2014_9637_MOESM1_ESM.docx]

**Table 1. PortionControl@HOME Intervention Elements, theoretical foundation, description and participation rates of elements and assignments.**

| Intervention elements & Theoretical foundation | Element description | Participation rates of intervention elements and assignments | |
| --- | --- | --- | --- |
| PortionSize@warenessTool |  |  |  |
| According to the Precaution Adoption Process Model, awareness is the first stage of health behavior change, and increasing awareness is considered a crucial first step in any health behavior change intervention [1]. It has been acknowledged that individuals' awareness of their current behavior and awareness about the recommendations are important prerequisites for behavior change. People who are unaware that they are engaging in unhealthy behaviors (e.g., selecting large portions) or do not have accurate knowledge about the desired behavior are unlikely to address the healthy behavior (e.g., selecting and consuming appropriate servings). | The PortionSize@warenessTool is an online interactive tool to increase awareness about the relationship between portion size and energy intake, recommended serving sizes and factors that contribute to the selection and intake of large food portions (i.e. value marketing). This element has been described in detail elsewhere, with previous results showing that using the tool resulted in significant improvements in portion size awareness [2]. | - Logged-in on the PortionSize@awarenessTool ^a^ | N = 126 (90.4%) |
| Portion Control Strategies |  |  |  |
| Self-regulation refers to all efforts to steer attention, emotions and behaviors to reach beneficial long-term goals (i.e. weight loss), even when there are short-term temptations (i.e. a nice cookie) or conflicting long-term goals [3]. In the context of controlling the amount of food consumed, self-regulation refers to efforts to control and maintain adequate selection and intake of the amount of food, thereby resisting or adapting temptations and situations by which one is triggered to overeat [4]. According to the health action process approach several assessments (action planning, coping planning and self-monitoring) can be used to improve actual behavioral change. For example, by action planning, individuals develop a mental representation of a suitable future situation (‘‘when’’ and ‘‘where’’) and a behavioral action (‘‘how’’ e.g. using the portion control strategy), which is expected to be effective to achieve the goal [5]. Coping planning helps individuals to define difficult situations (‘‘when’’ and ‘‘where’’) how to respond to overcome this barrier (how). An important predictor of weight loss maintenance suggested previously is self-monitoring of the changed behavior or weight loss [6, 7]. Self-monitoring consists of recording eating behavior and dietary intake so that people are aware of their current behaviors [7]. | Portion control strategies enable people to regulate the portion size they select and eat. An 8-minute video introduction outlined the aim of the strategies and presented several scenarios in which people are vulnerable to select and consume large food portions. An educational book delineated the influences of portion sizes and explored the behavioral strategies in more detail [8]. In order to improve participants’ initial and prolonged use of the portion control strategies, self-regulation assignments, such as action planning, coping planning and self-monitoring were incorporated as these strategies have been found effective in achieving changes in health behaviors [6, 7, 9, 10]. | *-* Watched 8-minute introduction video ^b^   - Partly - Totally   *-* Read educational book   - Partly - Totally   Fulfillment of the assignments:  *- Action planning assessment*   - In mind - Wrote down on assessment form   *- Coping planning assessment*   - In mind - Wrote down on assessment form   *- Self-monitoring assessment*   - In mind - Wrote down on assessment form | N= 9 (10.5%)  N= 63 (73.6%)  N= 29 (33.7%)  N = 55 (64%%)  N= 39 (46.4%)  N = 15 (17.9%)  N= 16 (19.0%)  N= 20 (23.8%)  N= 17 (19.8%)  N= 10 (11.6%) |
| Portion Control Cooking Class |  |  |  |
| Energy density is defined as the number of calories in a given weight of food. The higher the energy density, the more calories a meal of a given portion size contains. Lowering the energy density of a meal is an additional solution over and above portion size reduction. In doing so, one might be able to keep the same portion size, but lower the caloric content of the consumed portion [11-13]. Energy density can be reduced by for example adding more water rich ingredients (such as vegetables) or decreasing the fat content [14]. During each class, techniques to lower the energy density of meals were provided, peer discussions were stimulated and skills were trained by preparing meals. | A three bi-weekly cooking classes (3 hours each) that were led by dietician and health professionals (M.P.P. and E.V.) were offered. The portion control cooking classes provided demonstrations of 1) appropriate servings of common foods consumed and 2) preparation of meals lower in energy density During each class, techniques to lower the energy density of meals were provided, peer discussions were stimulated and skills were trained by preparing meals. | *- Participated in the Portion Control Cooking Class* ^a^   - *never* - *1 session* - *2 sessions* - *3 sessions* - *At least one session* | N= 28 (20.1%)  N= 21 (15.1%)  N= 34 (24.5%)  N= 56 (40.3%)  N= 111 (79.9%) |
| Portion Control Home-Screener |  |  |  |
| The modern food environment has been related to an increase in people’s energy intake and has therefore been associated with the raise in overweight and obesity prevalence [15]. An important setting that influences eating behavior and dietary intake is the home food environment [16]. The home food environment is the place where the retail food environment comes together with actual food intake [17]. In the Netherlands, approximately 81% of all meals consumed at home [18]. Moreover, the home food environment is fundamental in the development food preferences and consumption habits [19, 20]. According to the ANGELO-framework (ANalysis Grid for Environments Linked to Obesity) the physical home food environment which refers to what is present in the home [21]. | The ’Home-Screener’ is a screening-and feedback instrument developed to assist individuals to shape a home food environment that supports adequate portion control behavior. The Home-Screener was developed by the research team and was pilot tested among more than 90 households (unpublished work). Based on this pilot test, items that could not be assessed reliably were deleted and the Home-Screener was developed for its final form as used in this study. The Home-Screener identify aspects of the physical home food environment that influence the amount of food consumed [22-27]. It includes 20 questions on the amount of food available, the visibility and accessibility of foods, contextual food cues and the size of crockery and cutlery. As the Home-Screener was developed to eliminate surplus energy intake, the instrument targeted energy dense foods and beverages (e.g. snacks, sweets, sugar sweetened beverages). Participants could screen their home food environment indicate the supportiveness of the physical home food environment of adequate portion control behavior. Based on their screening, users were provided with feedback (i.e. which home environmental changes could be effective) and written assignments (i.e., writing down plans on what to change) to improve their home food environment in such a way that it stimulated adequate portion size selection and intake. | *- Home-Screener use* ^b^   - *Filled- out home screener (yes)* - *Read advice presented (yes)* | N= 41 (48.2%)  N= 64 (75.3%) |
| Portion Control Boosters |  |  |  |
| The portion control boosters were based on the theoretical approaches and frameworks of the four elements of the PortionControl@HOME intervention. | At the beginning of the summer (June 2012), the late summer (September 2012) and prior to the Christmas-holidays (December 2012) portion control Boosters were provided by email. Booster 1 contained information about reference serving sizes, extra tips to control portion size selection specified at typical summer events (e.g. during holidays) and provided some low-energy dense recipes for typical barbecue dishes. Booster 2 repeated all portion control strategies provided during the intervention period. Moreover, individuals were stimulated to monitor their current use of the strategies and to formulate new coping plans to stay on track. Booster 3 repeated the portion control strategies and home-environmental tips aimed at typical Christmas holiday temptations and appropriate servings of typical Christmas meals were presented. Participants were also encouraged to visit the PortionSize@warenessTool again. | No measures determined |  |

**References**

1. Weinstein ND. The precaution adoption process. *Health Psychol*. 1988; 7: 355-386.

2. Poelman MP, Steenhuis IH, de Vet E, Seidell JC. The development and evaluation of an Internet-based intervention to increase awareness about food portion sizes: a randomized controlled trial. *J Nutr Educ Behav*. 2013; 45(6): 701-707.

3. De Ridder D, de Wit J. *Self-regulation in health behavior*. Sussex: Wiley; 2006.

4. Poelman MP, de Vet E, Velema E, Seidell JC, Steenhuis IH. Behavioural strategies to control the amount of food selected and consumed. *Appetite*. 2014; 72: 156-165.

5. Gollwitzer PM. Implementation intentions—Strong effects of simple plans. *Am Psychol*. 1999; 54: 493-503.

6. Foster GD, Makris AP, Bailer BA. Behavioral treatment of obesity. *Am J Clin Nutr*. 2005; 82(1):230 s-5s.

7. Burke LE, Wang J, Sevick MA. Self-monitoring in weight loss: A systematic review of the literature. *J Am Diet Assoc*. 2011; 111(1):

92-102.

8. Steenhuis IHM, Poelman MP, Overtoom W. *Smartsize me Een slimme manier om maat te houden*. Schiedam: Scriptum; 2011.

9. Elfhag K, Rossner S. Who succeeds in maintaining weight loss? A conceptual review of factors associated with weight loss maintenance and weight regain. *Obes Rev*. 2005; 6(1): 67-85.

10. Wade DT. Goal setting in rehabilitation: An overview of what, why and how. *Clin Rehabil*. 2009;23(4): 291-295.

11. Ello-Martin JA, Ledikwe JH, Rolls BJ. The influence of food portion size and energy density on energy intake: Implications for weight management. *Am J Clin Nutr*. 2005; 82(1): 236 s-41s.

12. Kral TV, Rolls BJ. Energy density and portion size: Their independent and combined effects on energy intake. *Physiol Behav*. 2004; 82(1): 131-138.

13. Rolls B, Roe L, Meengs J. Reductions in portion size and energy density of foods are additive and lead to sustained decreases in energy intake. *Am J Clin Nutr*. 2006; 83(1): 11-17.

14. Rolls B, Barnett RA. *The volumetrics weight-control plan: feel full on fewer calories*. New York: HarperTorch;2005.

15. Brownell KD, Horgen KB. *Food Fight: the inside story of the food industry, America’s obesity crisis, and what we can do about it*. New York: McGraw Hill; 2004.

16. Rosenkranz RR, Drewaltowski DA. Model of the home food environment pertaining to childhood obesity. *Nutr Rev*. 2008; 66(3): 123-140.

17. Lin BH, Guthrie, J. & Frazao, E. (1999) Nutrient contribution of food away from home. In: America’s Eating Habits: Changes and Consequences (Frazao, E., ed.), pp. 213-242. AIB No. 750,U.S. Department of Agriculture, Economic Research Service, Washington, D.C

18. van Berlo I, Huijgen K, van Aalst M. Consumententrends. Centraal Bureau Levensmiddelenhandel (English translation: Food retail association) en EFMI business school 2011. Available from: http://www.supermarkt.nl/cblopleidingen/bronnen/Consumententrends2011.pdf (accessed March 2014).

19. Birch LL, Davison KK. Family environmental factors influencing the developing behavioral controls of food intake and childhood overweight. *Pediatr Clin North Am*. 2001; 48(4): 893-907.

20. Kral TVE, Rauh EM. Eating behaviors of children in the context of their family environment. *Physiol Behav*. 2010; 100(5): 567-573.

21. Swinburn B, Egger G, Raza F. Dissecting obesogenic environments: The development and application of a framework for identifying and prioritizing environmental interventions for obesity. *Prev Med*. 1999; 29(6 Pt 1): 563-570.

22. Wansink B. Environmental factors that increase the food intake and consumption volume of unknowing consumers. *Annu Rev Nutr*. 2004; 24: 455-479.

23. Campbell KJ, Crawford DA, Salmon J, Carver A, Garnett SP, Baur LA. Associations between the home food environment and obesity-promoting eating behaviors in adolescence. *Obesity*. 2007; 15(3): 719-730.

24. Chandon P, Wansink B. When are stockpiled products consumed faster? A convenience-salience framework of postpurchase consumption incidence and quantity. *J Marketing Res*. 2002; 39: 321-335.

25. Ferriday D, Brunstrom JM. How does food-cue exposure lead to larger meal sizes? *Br J Nutr*. 2008;100(6): 1325-1332.

26. Sobal J, Wansink B. Kitchenscapes, tablescapes, platescapes, and foodscapes—Influences of microscale built environments on food intake. *Environ Behav*. 2007; 39 (1): 124-142.

27. Wansink B, Sobal J. Mindless eating—The 200 daily food decisions we overlook. *Environ Behav*. 2007; 39: 106-123.
